# ULK1 phosphorylates Sec23A and mediates autophagy-induced inhibition of ER-to-Golgi traffic

**DOI:** 10.1186/s12860-017-0138-8

**Published:** 2017-05-10

**Authors:** Wenjia Gan, Caiyun Zhang, Ka Yu Siu, Ayano Satoh, Julian A. Tanner, Sidney Yu

**Affiliations:** 1 School of Biomedical Sciences, Shatin, N.T., Hong Kong, Special Administrative Region of China; 2 Department of Clinical Laboratory, The First Affiliated Hospital of Sun Yat-sen University, Guangzhou, Guangdong People’s Republic of China; 3 The Graduate School of Natural Science and Technology, Okayama University, Tsushima naka 3-1-1, Okayama, 7008530 Japan; 4 School of Biomedical Sciences, University of Hong Kong, 21 Sassoon Road, Pok Fu Lam, Hong Kong, Special Administrative Region of China; 5 Epithelial Cell Biology Research Center, The Chinese University of Hong Kong, Shatin, N.T., Hong Kong, Special Administrative Region of China

**Keywords:** Sec23, ULK1, Autophagy, COPII, ER exit sites

## Abstract

**Background:**

Autophagy is an inducible autodigestive process that allows cells to recycle proteins and other materials for survival during stress and nutrient deprived conditions. The kinase ULK1 is required to activate this process. ULK1 phosphorylates a number of target proteins and regulates many cellular processes including the early secretory pathway. Recently, ULK1 has been demonstrated to phosphorylate Sec16 and affects the transport of serotonin transporter at the ER exit sites (ERES), but whether ULK1 may affect the transport of other cargo proteins and general secretion has not been fully addressed.

**Results:**

In this study, we identified Sec23A, a component of the COPII vesicle coat, as a target of ULK1 phosphorylation. Elevated autophagy, induced by amino acid starvation, rapamycin, or overexpression of ULK1 caused aggregation of the ERES, a region of the ER dedicated for the budding of COPII vesicles. Transport of cargo proteins was also inhibited under these conditions and was retained at the ERES. ULK1 phosphorylation of Sec23A reduced the interaction between Sec23A and Sec31A. We identified serine 207, serine 312 and threonine 405 on Sec23A as ULK1 phosphorylation sites. Among these residues, serine 207, when changed to phospho-deficient and phospho-mimicking mutants, most faithfully recapitulated the above-mentioned effects of ULK1 phospho-regulation.

**Conclusion:**

These findings identify Sec23A as a new target of ULK1 and uncover a mechanism of coordinating intracellular protein transport and autophagy.

**Electronic supplementary material:**

The online version of this article (doi:10.1186/s12860-017-0138-8) contains supplementary material, which is available to authorized users.

## Background

Autophagy is an inducible lysosomal degradation process that recycles proteins and organelles to supply the cells with amino acids, lipids and energy for survival. This process is activated during stresses like amino acid starvation. mTORC1 complex regulates the signaling pathway leading to activation of autophagy by phosphorylating and inhibiting the kinase activity of ULK1 (Unc-51 like kinase 1). Upon nutrient deprivation, repression by mTORC1 is relieved and active ULK1 forms complex with mAtg13, FIP200 (or RB1CC1), and Atg101 [1–8], leading to autophagic activation. In yeast, ULK1 homologue Atg1 plays an instructive role in the formation of pre-autophagosomal structure (PAS) [9]. ULK1 phosphorylates downstream effectors to initiate autophagy. A downstream target of ULK1 has recently been identified. ULK1 is recruited to the Beclin-1-ATG14L-VPS34 complex via its interaction with ATG14L. Beclin-1 is activated by ULK1 phosphorylation and subsequently, the PI3 kinase activity of VPS34 stimulates the production of PtdIns(3)P needed for the formation and/or maturation of autophagosomes [10]. With broad effects of ULK1 in autophagy activation, it is likely that ULK1 may have other phosphorylation targets.

The vesicular transport process starts at the endoplasmic reticulum (ER). ER-derived COPII vesicles are formed by cytosolic protein factors collectively called COPII coat proteins, [11] consisting of Sar1, Sec23, Sec24, Sec13, and Sec31. The small GTPase Sar1, when bound to GTP, initiates COPII vesicle formation by recruiting the Sec23-Sec24 dimer to form the inner coat. Then, Sec13-Sec31 dimer is recruited to form the outer coat of a COPII vesicle. Sec23 is the GTPase-activating protein (GAP) for Sar1. Its GAP activity can be stimulated by Sec31. Sec24, the interacting partner of Sec23, determines the specificities of the cargo proteins in the ER to be incorporated into budding COPII vesicles. Sec23 recruits Sec13-Sec31 dimer to complete COPII vesicle formation. It is believed that formation of COPII vesicles adopts a similar mechanism in higher eukaryotes. Multiple homologs of COPII components are present in drosophila and mammalian cells, suggesting functional redundancy and complex regulation of cargo selection. In mammalian cells, components of the COPII coat present punctate structures that decorate the ER tubules under fluorescent microscopy. These structures are defined as ERESs, where budding of COPII vesicles occurs.

The connection between the early secretory pathway and autophagy has been previously reported but has, until recently, been limited to how the early secretory pathway mechanistically contributes to the formation of autophagosomes [12–16]. In yeast *Saccharomyces cerevisiae*, mutants of Sec12, Sec23 and Sec24 were also found impaired in autophagy [17]. Furthermore, the TRAPPIII (*Tra*nsport *p*rotein *p*article III) complex has been implicated to function in autophagy in yeast [18, 19]. The TRAPP complex was originally identified as a tethering factor for COPII vesicles but a distinct form, TRAPPIII, has been found to activate Ypt1 and then recruit Atg1 to preautophagosomal structure (PAS) in yeast. Recently, Sec23 and Sec24AB were, reportedly, required to form a non-membranous, lipid droplet-like structure called Sec body when the cells were starved with amino acids in Drosophila S2 cells [20]. Such structure was thought to be a protective mechanism for preserving the secretory pathway during nutrient stress, so that re-building of functional secretory pathway is possible after the stress is relieved.

We believe it is also advantageous for mammalian cells with elevated autophagy to slow down cellular secretion at the same time because such coordination is consistent with the purpose of nutrient and energy conservation. To investigate whether autophagy causes any change to the early secretory pathway in mammalian cells, we found that ERES morphology is different in cells undergoing active autophagy and protein intracellular transport is inhibited at the ERESs. This inhibition is, at least in part, caused by the phosphorylation of Sec23A at serine 207 and Threonine 405 by ULK1. ULK1 phosphorylation reduces the interaction between Sec23A and Sec31A. These results explain the change of ERES morphology and the reduced cellular secretion in cells undergoing active autophagy.

## Methods

### Reagents and antibodies

Monoclonal antibodies against Sec31A (Cat. No. 612350) and against phospho-Ser/Thr (Cat. No. 612548) were purchased from BD Biosciences. Rabbit polyclonal antibodies against LC3B (Cat. No. L7543), against Calnexin (Cat. No. C4731), and monoclonal antibody against FLAG (Cat. No. F1804) were purchased from Sigma. Monoclonal antibody against ERGIC-53 was obtained from Dr. Hans-Peter Hauri (University of Basel, Switzerland) [21]. Monoclonal antibody against GFP was purchased from Roche (Cat. No. 11814460001). Monoclonal antibody against c-Myc (9E10) and TRITC-conjugated 9E10 were purchased from Santa Cruz Biotechnology (Cat. No. sc-40). HRP or Alexa Fluor conjugated secondary antibodies were obtained from sources previously described [22, 23].

Rapamycin (Santa Cruz, sc-3504) and MHY1485 (Sigma, SML0810) were dissolved in DMSO at 5 mM and 10 mM as stock, respectively.

### Plasmids

FLAG-FIP200, pCMV-Myc-ULK1 wild type and kinase dead mutant (M92A) were purchased from Addgene [4, 7]. pCMV-Myc-His-Sec23A were constructed by cutting the cDNA containing Sec23A sequence from pCMV-Myc-Sec23A [22], and subcloned into pCMV-Myc-His vector at XhoI and NotI sites. pEGFP-Sec23A was constructed similarly but on XhoI and BamHI sites. The pCMV-Myc-His vector was modified from pCMV-Myc by in-frame insertion of a linking containing six histidine between the c-Myc tag and the multiple cloning site. pCMV-Myc-His-Sec31A was constructed from pCMV-Myc-Sec31A by the same method as above. Other plasmids expressing Sec13, Sec24 and Sar1b were previously constructed [22].

Sec23A mutants were constructed by PCR-based megaprimer site-directed mutagenesis. For each mutagenesis, the mutagenesis primer in reversed orientation was paired with a forward primer annealed to the 5′ of Sec23A sequence for PCR amplification, and the mutagenesis primer in forward orientation was paired with reversed primer of the 3′ of Sec23A. The resulting PCR fragments containing the desired mutation were mixed for second PCR amplification to generate full length Sec23A mutant sequence. The mutations were all confirmed by DNA sequencing.

### Cell Culture

Hela, HEK293T, HEK293 and COS cell lines were obtained from the American Type Culture Collection (ATCC) and maintained in Dulbecco’s Modified Eagle’s Medium (DMEM) supplemented with 10% fetal bovine serum (FBS). Transfections of DNA plasmids were carried out by PEI mediated delivery according to procedures previously described. [23] In experiments that needed to deprive the cells with amino acids to activate autophagy, the cells were washed with PBS several times and then incubated with EBSS (Life Technology) for one to two hours before further processing. Autophagy was also induced by rapamycin at 0.5 μM in growth medium for three hours. MHY1485 was used to inhibit autophagy at 5 μM for three hour incubation.

For experiments measuring VSVG transport, cells seeded onto glass coverslips were transfected with DNA plasmid expressing GFP-VSVG-tsO45 (a temperature sensitive mutant of the Vesicular Stomatitis Virus G glycoprotein). The transfected cells were incubated at 39.5 °C overnight to allow expression of VSVG. The next day, the cells were shifted to 32 °C permissive temperature so that VSVG could refold and exit the ER and transport along the secretory pathway. The cells were fixed and stained at the indicated time after 32 °C temperature shift.

### Immunoprecipitation

For immunoprecipitation, cells transfected with indicated mammalian expression plasmids were harvested by scraping in RIPA (150 mM NaCl, 20 mM Tris pH 8.0, 0.01% NP-40) supplemented with proteinase inhibitor cocktail (no EDTA, Roche). After centrifugation, the supernatant was collected into a new tube. Then, protein A-Sepharose beads coupled with Mouse anti-Myc or anti-GFP antibody were added into the supernatant. The mixture was incubated at 4 °C for 3 h. Then, beads were collected and washed 4 times with RIPA. Lysate and immunoprecipitated samples were heated at 90 °C for 5 min before loading on SDS-PAGE gel. For His-tag pull down, cell lysate was prepared as described above. Then, Cobalt resin was added into the supernatant. The mixture was incubated at 4 °C for 3 h. Then, beads were collected by centrifuge and washed 4 times with RIPA plus 4 mM imidazole. Samples were also heated at 90 °C for 5 min before loading on SDS-PAGE gel.

### Kinase assay

HEK293T cells grown 15-cm dish was transfected with 30 μg of pCMV-Myc-His-Sec23A for 24 h. The cells were trypsinized and collected by centrifugation. The cell pellet was washed once with PBS and resuspended in 3 ml of PBS containing protease inhibitor cocktail. The resuspension was frozen and thawed once before subjected to sonication. After sonication, the sample was centrifuged at 10,000 g for 10 min at 4 °C. The supernatant was collected into a new tube and incubated with approximately 100 μl of Cobalt resin (HisPur Cobalt Resin, Thermo Fisher, 89,964) at 4 °C for 3 h. The resin was collected and washed 4 times with PBS containing 4 mM imidazole on ice. The Myc-His-Sec23A protein was eluted from the resin by 0.5 ml of PBS containing 400 mM of imidazole and incubating on ice for 5 min with occasional inversions. The eluted fraction was collected after brief centrifugation and dialyzed against PBS to remove imidazole.

Precipitated ULK1, Sec23 wild type and/or mutants were incubated at 30 °C for 15 min in kinase assay buffer (10 mM Tris-Cl, pH 7.5, 150 mM NaCl, 10 mM MgCl_2_, 5 mM dithiothreitol) supplied with 25 μM ATP, with occasional mixing. The samples were subjected to SDS-PAGE and immunoblotting using Mouse anti-Myc and anti-pSer/Thr antibodies as in experiments shown in Figs. 4c and 5b. For determination of ULK1 phosphorylation sites, phosphorylated and non-phosphorylated Sec23 were generated by co-transfecting HEK293T cells with Myc-His-Sec23A and ULK1 or with ULK1 KD mutant, respectively. Sec23A were purified from cell lysates by cobalt resin as described above. Then the samples were run on SDS-PAGE gel and protein bands corresponding to the expected molecular weight of Myc-His-Sec23A based on Coomassie blue staining was cut and sent to mass spectrometry analysis. BGI (Shenzhen, China) determined the phosphorylation sites by Liquid chromatography (LC)-tandem quadrupole mass spectrometry. Putative phosphorylation sites shown in Fig. 5a were identified from the peptide by Mascot Phosite. PhosphoRS Probability indicated the probability of amino acid residues indicated are the ones containing the phosphate. Human Sec23A sequence (Accession number Q15436.2, GI:143,811,354) from NCBI was used as reference.

### Fluorescence microscopy and quantification

Cells grown on 12 mm cover slips (Thermo) were washed with PBS 3 times to remove medium completely, followed by fixation with 4% polyformaldehyde (PFA) in PBS for 15 min. Then, the cells were rinsed in 1% BSA in PBS and with PBS plus 0.1% Triton X-100 for 5 min for permeabilization. Then, cells were blocked in 1% BSA in PBS for 30 min. 12–15 μl diluted primary antibody (usually in 1–2 μg/ml) in PBS plus 1% BSA was applied to the cells at room temperature for 60 min. After being washed with PBS plus 1% BSA for 3 times, the cells were incubated with 12–15 μl diluted Alex Fluor conjugated secondary antibody in PBS plus 1% BSA at room temperature for 60 min. After washing with PBS plus 1% BSA for 3 times, the cover slips were rinsed in autoclaved water and mounted on glass slides using fluoromount-G (Thermo). Slides were air dried and stored at −20 °C or observed under the microscope. Confocal images were acquired in a Leica SP5 microscope with 63 X objective lens. For measurement of the percentage of VSVG retaining in ERES, the number of GFP-VSVG (green), and Sec31A (red) measured by Manders’ coefficients (faction of GFP-VSVG overlapping with Sec31A) from ImageJ v1.49 JaCoP plugin.

## Results

### ULK1 interacts with Sec23A

**Fig. 1:**
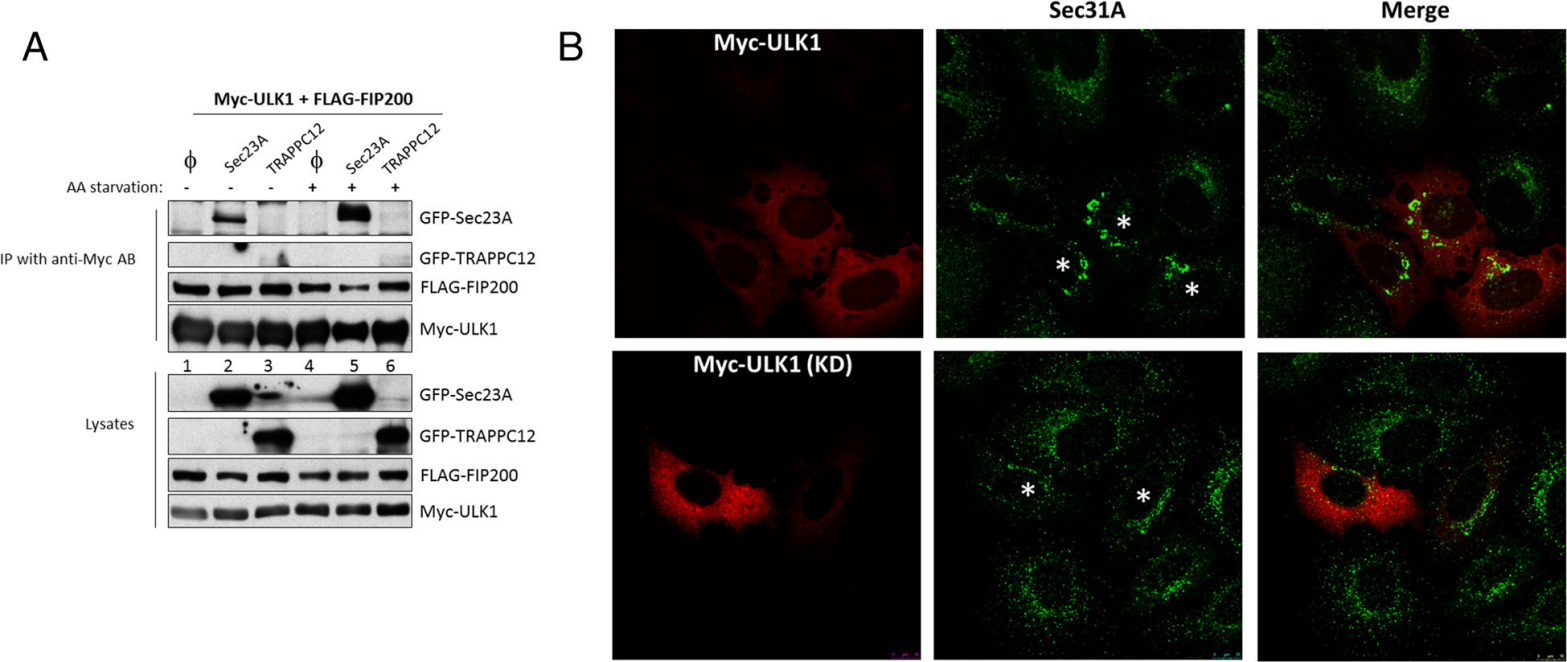
ULK1 interacts with Sec23A and affects ERES morphology. **a** Sec23A but not TRAPPC12 was pulled down by ULK1 in co-immunoprecipitation experiment. Myc-ULK1, FLAG-FIP200 were co-transfected with the indicated GFP fusion DNA constructs. Myc-ULK1 was immunoprecipitated with anti-c-Myc antibody and the presence of GFP-Sec23A or GFP-TRAPPC12 was determined. **b** ULK1 affects the morphology of ERES. Myc-ULK1 wild type or kinase dead (KD) mutant were co-transfected with GFP-Sec23A into Hela cells. ULK1 transfected cells were determined by immunostaining of TRITC conjugated anti-Myc antibody. ERES was detected with Sec31A staining. *Asterisks* in the Merge pictures indicate cells transfected with ULK1

Recently it was reported that the substrate of TRAPPIII, Rab small GTPase Ypt1, interacted with Atg1 (the yeast ortholog of ULK1) for activation of autophagy at the PAS [24]. Because components of TRAPPIII, such as TRAPPC12 (a.k.a. TTC-15) have been previously demonstrated to be part of the autophagy signaling network in mammals [25], we were interested in finding out if some TRAPPIII components physically interacted with those of the ULK1 complex by co-immunoprecipitation. Unfortunately, we did not observe consistent and physiologically meaningful interactions in this investigation (data not shown). However, in one of the interaction studies, we fortuitously observed that Sec23A interacted with ULK1. In this experiment, TRAPPC12 was not co-precipitated with ULK1 from cells grown in complete growth medium (lanes 3 and 6, second panel, Fig. 1a), but Sec23A interacted with ULK1 strongly and the interaction drastically increased when the cells were first deprived with amino acid to induce autophagy before co-immunoprecipitation (lanes 2 and 5, top panel, Fig. 1a). This result prompted us to determine if ULK1 caused any morphological changes to the ERES in cells. We transfected ULK1 into Hela cells and found that ULK1 caused clustering of ERES into large fluorescent puncta (Fig. 1b, upper panels). As control, overexpressing the kinase-deficient (KD) mutant of ULK1 could not cause such change (Fig. 1b, lower panels). The effect of ULK1 on ERES was also observed in HEK293 cells (data not shown), suggesting this was a general effect of ULK1. ULK1 may act by itself because this effect could not be subdued by depletion of hATG13 (data not shown).

### Autophagy causes morphological change in ERES

**Fig. 2:**
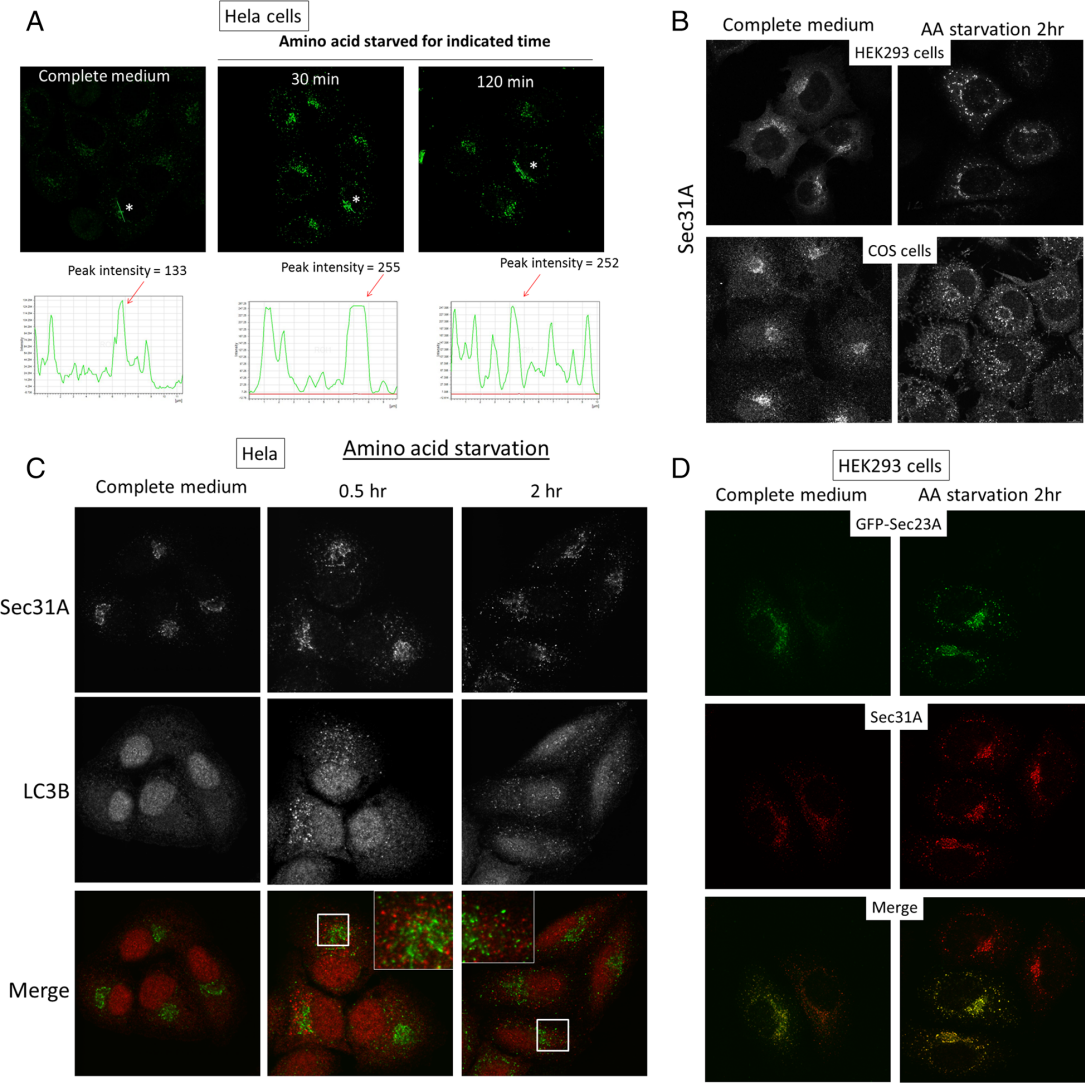
Autophagy changes the morphology of ERES. **a** Hela cells were cultured in growth medium or amino acid starved in EBSS to activate autophagy for indicated time. Cells were stained with ERES by anti-Sec31A antibody. Fluorescence intensity profiles of the ERES of the cells marked with *asterisks* were quantified. A line was drawn from a cell chosen in the image to generate fluorescence pixel intensity. **b** ERES in HEK293 (top) and COS cells (*bottom*) in complete growth medium or in EBSS (AA starvation). **c** Hela cells incubated with complete medium or EBSS for amino acid starvation for 0.5 or 2 h were stained with ERES (Sec31A, green in merge) and autophagosomes (LC3B, red in merge). **d** HEK293 cells were transfected with GFP-Sec23A (*green*). Transfected cells were cultured in growth medium or EBSS for 2 h before fixation and staining with Sec31A (*red*)

Since ULK1 was an important kinase regulating autophagy activation, we investigated if active autophagy altered the ERES similar to overexpression of ULK1 in a number of commonly used laboratory cell lines. Amino acid starvation is a strong inducer for autophagy. When Hela cells were activated for autophagy by deprivation of amino acids, the ERES changed from a perinuclear staining pattern with low level of cytosolic signal throughout the cells to a more punctate staining pattern and aggregated into fewer but brighter fluorescence dots at the perinuclear region (Fig. 2a). To precisely illustrate the increase in ERES fluorescence, we randomly picked a cell from each image and measured the intensity of the fluorescence pixels by drawing a line across the area most concentrated with ERES (asterisks, Fig. 2a). The peak fluorescence intensity of the ERES under amino acid starvation was approximately twice as strong as the one under normal growth medium (Fig. 2a, bottom panels). An overall change at ERES pixel intensities in boxed areas, instead of a line drawn across the ERES, was presented in Additional file 1: Figure S1. Such change in the ERES morphology was reminiscent of the effect of ULK1 overexpression except that the extent of concentration was milder due to a lower ULK1 activity at endogenous level. Autophagy-induced morphological change of the ERES was also observed in HEK293 and COS cells (Fig. 2b). The concentration of ERES signals into fewer and brighter fluorescence puncta during active autophagy was reversible upon re-introduction of growth medium to the starved cells (Additional file 2: Figure S2A). The increased fluorescence puncta of Sec31A after autophagy activation were not extensively colocalized with LC3 puncta (Fig. 2c). We were also concerned that Sec31A might not truly represent the status of ERES as an ERES marker because ULK1 interacted with Sec23A, not Sec31A. Therefore, we investigated the status of Sec23A and Sec31A simultaneously after autophagy activation. In this experiment, GFP-Sec23A was first transfected before the cells were subjected to amino acid starvation to activate autophagy. Immunofluorescence staining of Sec31A did not reveal reduced co-localization with the transfected GFP-Sec23A (Fig. 2d). This data suggested that, at steady state, both the outer-layer (Sec23/Sec24) and the inner layer (Sec13/Sec31) of the COPII coat are subjected to the same change caused by elevated autophagy. We confirmed that an inhibitor of autophagy, MHY1485, was able to modulate the morphological change to ERES induced by amino acid starvation and that another activator of autophagy, rapamycin, could also increase the intensity of ERES puncta (Additional file 2: Figure S2B).

### Autophagy and ULK1 overexpression inhibits protein traffic at ERES

**Fig. 3:**
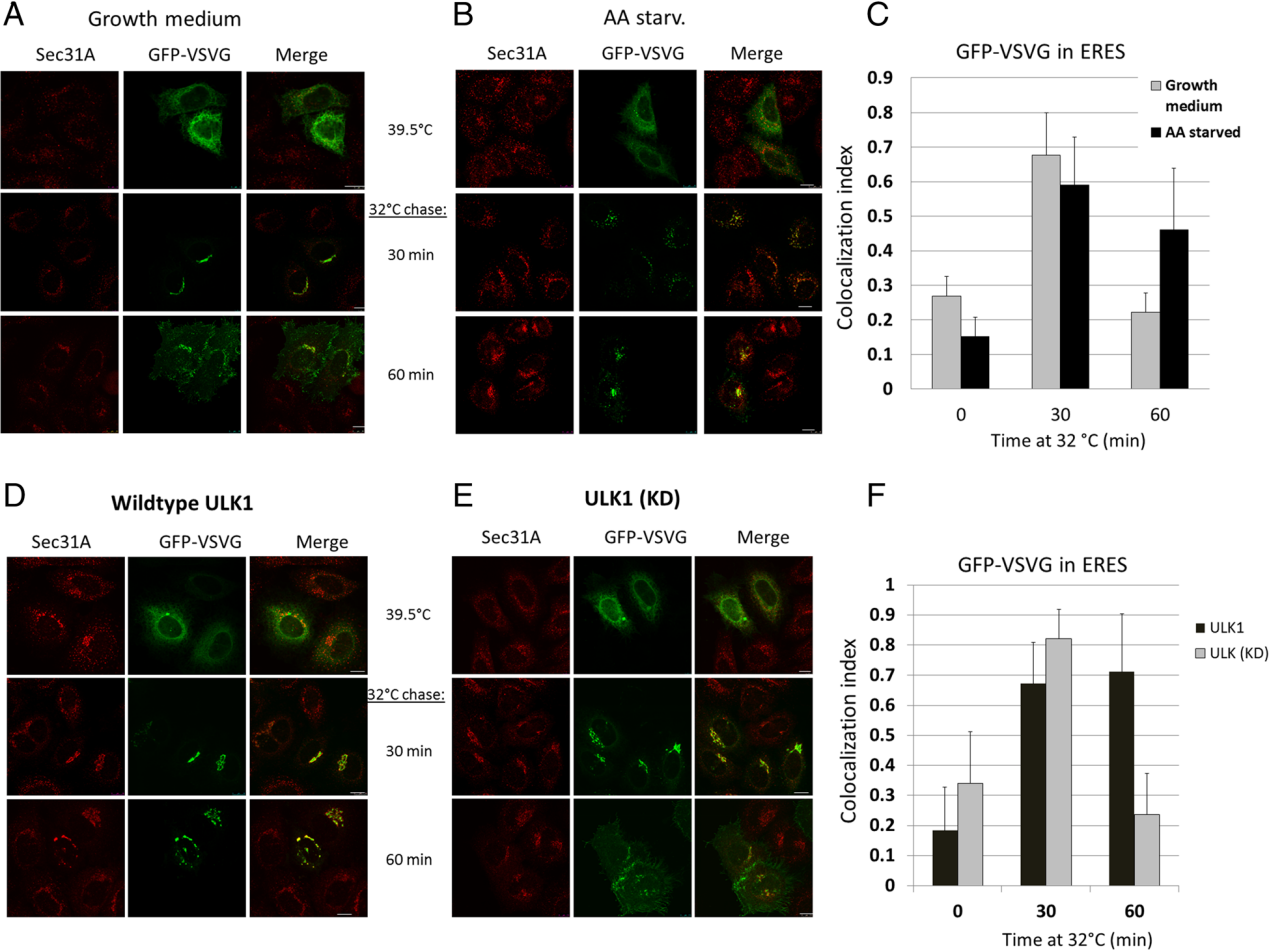
Autophagy and ULK1 caused retention of transport marker VSVG in ERESs. **a** Hela cells were transfected with GFP-VSVG-tsO45 and the cells were kept at 39.5C overnight. Then the cells were cultured in (**a**) normal growth medium or (**b**) induced with autophagy by amino acid starvation for 2 h before the incubation temperature was shifted to 32 °C for the indicated time to chase the VSVG out of the ER. **c** Quantification of the ERES-associated VSVG signals. The fractions of VSVG signal in ERES at different time points after 32 °C chase were measured by Manders Coefficients. Number of cells ≥10; Error Bars = S.D. **d** Hela cells were transfected with GFP-VSV-G tsO45 and Myc-ULK1 or (**e**) its kinase dead mutant at 1:4 ratio and the cells were kept at 39.5 °C. After being chased at 32 °C for different times as indicated, the cells were fixed and stained with Sec31A for ERES. **f** Quantification of the amount of ERES-retained VSVG signal in ULK1 or kinase dead mutant. The fractions of VSVG signal in ERES at different time points after 32 °C chase were measured by Manders Coefficients. Number of cells ≥ 8; Error Bars = S.D., *p* < 0.05

Because of the drastic changes at the ERES upon autophagy activation or ULK1 overexpression, we investigated if protein secretion was affected by in cells with elevated autophagy. By measuring the rate of secretion of the alkaline phosphatase activity in Hela cells stably transfected with a recombinant secreted form of alkaline phosphatase (SEAP), we found that secretion was reduced by autophagy activated by rapamycin and amino acid starvation (Additional file 3: Figure S3). To determine exactly where protein trafficking was affected, we monitored GFP-VSVG-tsO45 in cells cultured with growth medium and those activated with autophagy by amino acid starvation (Fig. 3a-b, respectively). At 39.5 °C, VSVG protein was misfolded and remained in the ER, and therefore, hardly any VSVG signal was colocalized with ERES marker Sec31A in both conditions (top two panels, Fig. 3a-b). When incubation temperature was shifted to 32 °C for 30 min, a majority of the VSVG signal had moved to the Golgi, and therefore, partially colocalized with Sec31A (middle two panels, Fig. 3a-b). At 60 min after temperature shift, VSVG signal remained associated with ERES marker Sec31A in cells activated with autophagy by amino acid starvation, whereas majority of the VSVG signal had reached the plasma membrane in cells incubated with growth medium (Fig. 3b-c). Retention of VSVG signal at ERES by active autophagy was also observed when the same experiment was performed using HEK293 cells (Additional file 4: Figure S4). Quantification of VSVG signal colocalized with ERES marker clearly demonstrated that elevated autophagy caused retention of VSVG at ERES (Fig. 3c). Since phosphorylation of Sec23A by ULK1 might be the reason of reduced traffic, we investigated this possibility by co-expressing GFP-VSVG-tsO45 with ULK1 or its kinase deficient mutant and then determined the trafficking of VSVG at the indicated time point. ULK1, but not its kinase deficient mutant, caused retention of VSVG at the ERES after the cells were shifted to 32 °C permissive temperature for one hour (Fig. 3d-e). A quantification of the VSVG signal was still highly colocalized with ERES marker Sec31A due to ULK1 60 min after temperature shift (Fig. 3f). At this time point, the kinase deficient mutant of ULK1 did not block VSVG transport and majority of the VSVG signal had reached the plasma membrane (Fig. 3b-c).

### ULK1 phosphorylates Sec23A at Serine 207 and Threonine 405

**Fig. 4:**
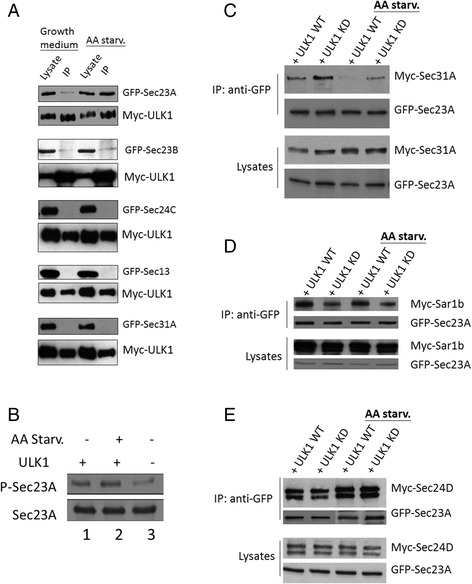
ULK1 phosphorylates Sec23 and changes its binding to other COPII components. **a** Myc-ULK1 WT was co-transfected with indicated COPII components (GFP-tag) pairwise in HEK293T cell. ULK1 was immunoprecipitated by anti-Myc antibody and the presence of co-precipitated COPII components were detected by blotting with anti-GFP antibody. **b** Myc-ULK1 and Myc-Sec23A were separately overexpressed and purified from HEK293T cells by immune-isolation using anti-Myc antibody. Some of ULK1 transfected cells were put under amino acid starvation to further activate the ULK1 kinase activity. Beads loaded with ULK1 and Sec23A protein were mixed to allow ULK1 to phosphorylate Sec23A in vitro. Phosphorylated Sec23a was detected by Mouse anti-serine/threonine antibody. Total Sec23a was detected by Rabbit anti-Sec23a antibody. **c-e** HEK293T cells were transfected with the indicated plasmids and treated by EBSS or complete medium prior to harvest. AA star. Means amino acid starvation. After harvest, Sec23A was immunoprecipitated and detected by using monoclonal anti-GFP antibody. Sec31A (**c**), Sar1b (**d**) and Sec24D (**e**) were detected by mouse anti-Myc antibody

**Fig. 5:**
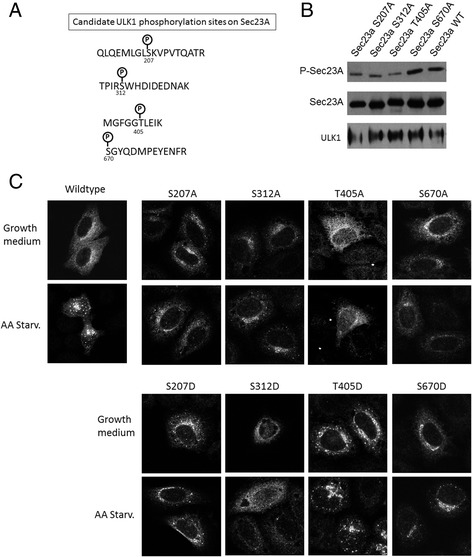
Identification of ULK1 phosphorylation sites on Sec23A. **a** Candidate ULK1 phosphorylation sites on Sec23A. **b** Various phospho-deficient mutants were tested for ULK1 phosphorylation. This experiment was similar to that in Fig. 3b with ULK1 activated, but in addition to wild type Sec23A, four alanine mutants of Sec23A were also used. **c** Phospho-deficient and phospho-mimicking mutants of Sec23A were analyzed for their morphology. Hela cells were transfected with wild type or indicated mutants of Myc-Sec23A and the Myc-Sec23A mutants were stained with anti-c-Myc antibody.Amino acid starvation was induced to cell for 2 h before fixation and staining (*lower rows*). Cell in the non-starved condition was cultured in complete growth medium before fixation (*upper rows*)

We tested if ULK1 also interacted with other subunits of the COPII coat. Myc-ULK1 and individual COPII components as GFP fusion constructs were co-expressed in cells and the presence of COPII subunits in the anti-Myc precipitation was determined by immunoblotting (Fig. 4a). We found that Sec23A, but not Sec23B, was the only COPII subunit that interacted with ULK1. We then tested if ULK1 directly phosphorylated Sec23A by expressing and isolating these proteins individually by immunoprecipitation and performed in vitro kinase assay. The reaction product was run on SDS-PAGE and phospho-Sec23 was determined by immunoblotting using an antibody specific to phospho-serine/threonine. In the absence of ULK1, the Sec23A isolated from cells was phosphorylated at a basal level (lane 3, Fig. 4b), probably by other kinases, such as casein kinase I. [26] However, when ULK1 was included in the kinase reaction, a drastic increase in phospho-Sec23A was detected (lane 1, Fig. 4b). A slightly further increase in phospho-Sec23A was observed in a reaction that included ULK1 isolated from cells that were previously activated with autophagy by amino acid starvation (lane 2, Fig. 4b). After we established that ULK1 directly phosphorylated Sec23A, we investigated how such phosphorylation would affect the function of Sec23A. To address this, we tested the interactions between Sec23A and Sar1b, Sec24D and Sec31A by co-immunoprecipitation under the influence of ULK1 activity (or with ULK1 kinase deficient mutant as negative control) (Fig. 5c-e). From these experiments, we observed that the interaction between Sec31A and Sec23A was inhibited by ULK1 and amino acid starvation further weakened the interaction (Fig. 4c). In contrast, kinase deficient ULK1 mutant could no longer inhibit Sec23A-Sec31A interaction. Sec23A-Sar1b interaction was slightly higher when co-expressed with ULK1 than ULK1 KD mutant. However, this effect was not exaggerated by amino acid starvation (Fig. 4d). Lastly, the interaction between Sec23A and Sec24D was not affected by ULK1 (Fig. 4e). Together, these results demonstrated that ULK1 bound to and phosphorylated Sec23A, and in turn, the interaction between Sec23A and Sec31A was weakened.

Having established ULK1 phosphorylated Sec23A, we tried to identify the site(s) of phosphorylation. Sec23A protein was purified and subjected to ULK1 phosphorylation in vitro, and then the sample underwent mass spectrometry identification of phosphorylated peptide sequence. Four candidate phosphorylation sites were identified and listed in Fig. 5a. Serine 207, serine 312, threonine 405, and serine 670 were suggested putative phosphorylation sites in ULK1 treated sample. We constructed phosphorylation deficient mutants by changing these residues to alanine individually and tested if ULK1 could still phosphorylate the mutant Sec23A. As shown in Fig. 5b, the Sec23A mutant proteins were expressed to a level similar to the wildtype protein, suggesting the mutation did not invoke degradation. The extent of phospho-Sec23A, however, varied significantly. S207A and T405A, and less consistently S312A, mutations all reduced the level of phospho-Ser/Thr of the protein, indicating they were bona fide ULK1 phosphorylation sites. S670 remained similar to wildtype Sec23A, suggesting this residue was not subjected to ULK1 phosphorylation. Next, we expressed the phosphorylation deficient mutants (i.e. Ser/Thr to Ala) and phospho-mimicking mutants (i.e. Ser/Thr to Asp) in cells to determine if any of these mutants recapitulated the change in ERES morphology caused by autophagy. Both S207A and T405A caused the mutant Sec23A protein slight more cytosolic even though the transfected cells were induced with autophagy by amino acid starvation (Fig. 5c). Their corresponding phopho-mimicking mutants, S207D and T405D presented highly punctate fluorescence signals similar to the ERES under active autophagy, and further increase in the fluorescence puncta was observed when the cells were activated for autophagy by deprivation of amino acids (Fig. 5c). The S207 and T405 mutants were also studied in COS cells and their expression pattern generally agreed with the results in Fig. 5 (Additional file 5: Figure S5). S312A and to S670A mutants could not block autophagy-induced ERES puncta, and therefore, we did not follow up on these mutations further. In summary, S207 and T405 are two residues that were phosphorylated by ULK1 and caused changes to the ERES morphology upon autophagy activation.

### Serine207 of Sec23A is important for Sec31A binding

**Fig. 6:**
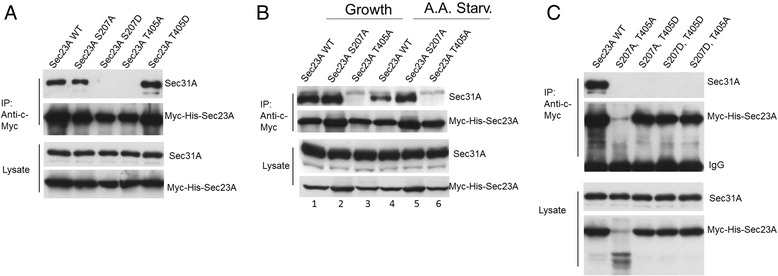
The interactions between the ULK1 phosphorylation mutants of Sec23A and endogenous Sec31A. **a** S207 and T405 mutants of Sec23A were tested for their interaction with Sec31A in normal growth condition. The indicated Myc-His-Sec23A mutants were transfected into cells and tested for their ability to bring down endogenous Sec31A in co-IP experiment. **b** Wildtype Sec23A, S207A and T405A mutants were tested for the interaction with Sec31A in growth medium or in amino acid starved medium EBSS. **c** Combinations of S207 and T405 double mutants were tested for the interaction with Sec31A. Overexpression of the indicated protein was carried in HEK293T cells, followed by immunoprecipitation of Myc-His-Sec23A by anti-Myc antibody. Co-precipitated endogenous Sec31A was detected by anti-Sec31A antibody

Since ULK1 phosphorylation weakened the interaction between Sec23A and Sec31A (Fig. 4c), we tested if mutating the residues S207 and T405 could recapitulate this change. Myc-His-Sec23A mutants were transfected and immunoprecipitated with anti-c-Myc antibody, and then the amount of Sec31A from endogenous source co-precipitated with Myc-His-Sec23A was determined. Mutation to S207 most appropriately mimicked the effect of ULK1 phosphorylation as S207A mutation showed normal Sec31 binding whereas S207D drastically reduced the interaction with Sec31A (Fig. 6a). Unexpectedly, T405 mutations did not behave as we expected. The phospho-deficient T405Abound very poorly to Sec31A but the phospho-mimicking T405D interacted with Sec31A normally. We tested how Sec31A interacted with S207A and T405A mutants during autophagy. As shown in Fig. 6b, wildtype Sec23A and S207A could pulldown endogenous Sec31A in cell cultured in growth medium, but the interaction between wildtype Sec23A and Sec31A was reduced when the transfected cells were incubated in amino acid starved medium for 2 h before subjected to immunoprecipitation (lane 4, Fig. 6b). This result was consistent with the effect of ULK1 overexpression in Fig. 4c. However, the S207A mutant completely abrogated this property: The extent of interaction between S207A and Sec31A under amino acid starved condition was the same as that in growth medium (lanes 2 and 5, Fig. 6b). We constructed double mutations at these residues and determine if their binding to Sec31A was affected. All combination of double mutants showed significant reduction in Sec31A binding compared to wildtype Sec23A (Fig. 6c). However, in this experiment, the S207A, T405A double mutant suffered from very extensive protein degradation, making the interpretation of its ability to bind to Sec31A unreliable by this experiment. These interaction studies were repeated with co-transfection of Myc-His-Sec23A and GFP-Sec31A and came up with the same conclusion (Additional file 6: Figure S6).

### Sec23A mutants block the formation of ER-Golgi intermediate compartments (ERGIC)

**Fig. 7:**
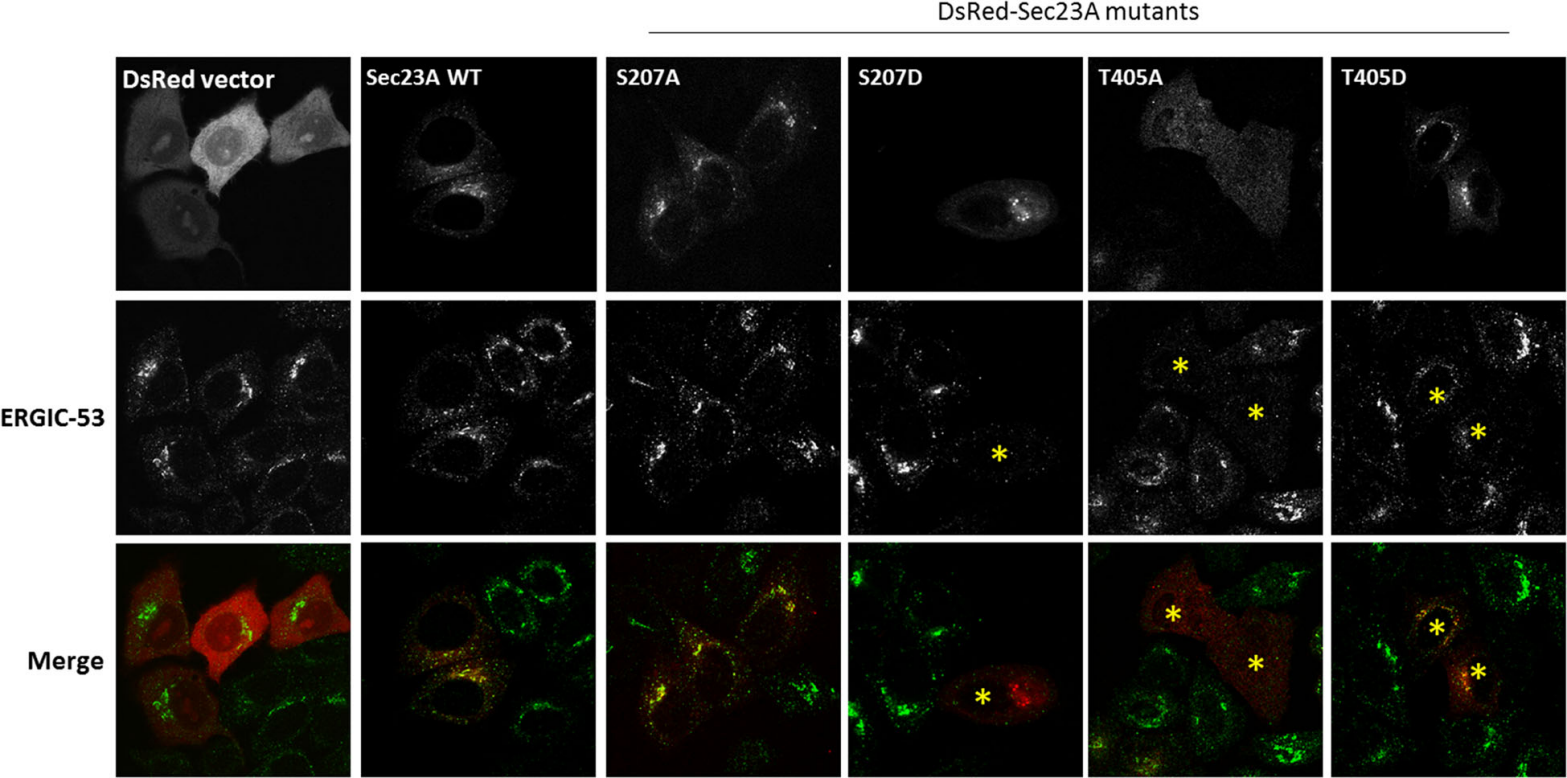
Expression of the Sec23A mutant compromised the integrity of ERGIC. Various indicated Sec23A mutants in DsRed vector were transfected to HeLa cells and the status of ERGIC was investigated by staining with marker ERGIC-53. DsRed and ERGIC-53 signals are pseudo-colored in *red* and *green*, respectively, in the merge panel (*bottom*)

To determine if overexpression of the Sec23A mutants affected ER to Golgi traffic, we initially investigated if the transport of VSVG was affected in this part of the secretory pathway but realized that overexpression of wildtype Sec23A could block VSVG transport (data not shown). This is probably due to the intrinsic GAP activity of Sec23A toward Sar1. Therefore, we, instead, investigated if the status of the ERGIC was changed (Fig. 7). Overexpression of DsRed vector or DsRed fused with wildtype Sec23A did not disrupt the morphology of ERGIC. However, Sec23A mutants S207D, T405A, and to a lesser extent T405D reduced the immunofluorescence staining signals of ERGIC-53 (asterisks, Fig. 7). This result strongly suggests ER-to-Golgi is inhibited by expression of the Sec23A mutants, impairing the biogenesis of the intermediate compartments. The ability of S207D and T405A to block ERGIC biogenesis also correlates with their loss of binding toward Sec31A.

## Discussion

Emerging evidence suggested connections between ERES and autophagosomes happened by multiple mechanisms [20, 27–31]. In this study, we have elucidated autophagy-activating kinase ULK1 phosphorylates Sec23A at residues serine 207 and threonine 405 and thereby, reduces traffic at ERES. Amino acid starvation-induced autophagy has been demonstrated to cause the formation of Sec body from ERES in drosophila S2 cells. While we hesitate to conclude the observed increase in fluorescence intensity of ERES in actively autophagic cells is equivalent to the Sec body found in S2 cells, it is clear that autophagy also exerts an influence on the early secretory pathway in mammalian systems [28, 29]. We believe this is a mechanism to coordinates cellular secretion and autophagy, with the ultimate goal of conserving nutrients (i.e., proteins are no longer secreted out of the starved cells). The change in ERES fluorescence puncta is not directly related to the role of ERES as membrane source for autophagosome biogenesis because we did not observe extensive colocalization of ERES with autophagosomes (Fig. 2C). However, it should be noted that signals of RFP-LC3 and GFP-Sec31A were quite frequently observed to be in close proximity by live cell imaging (data not shown), suggesting exchange of materials between these membrane compartments must occur. ULK1 has also been shown to phosphorylate Sec16A and its activity is required for normal morphology of ERES [28]. It was concluded that ULK1 had non-canonical function in regulating the secretion of Sec24C-specific cargos, e.g., serotine transporter (SERT). Conceptually different from a non-canonical function suggested by Joo et al. [28], here we provide evidence that general secretion at the level of ERES is inhibited by elevated ULK1 activity during active autophagy. Second, we found that secretion of two general transport markers, VSVG and SEAP [32, 33], rather than Sec24C-specific cargos, was inhibited during autophagy.

COPII vesicle formation is affected by ULK1 phosphorylation of Sec23A. In mammals, Sec23 has two homologs, Sec23A and Sec23B. We found that Sec23B interacted with ULK1 poorly, if at all (Fig. 3). No equivalent residue of S207 can be found in Sec23B. Overexpression of GFP-Sec23B was only partially colocalized with Sec31A and often presented reticulon pattern of expression rather than the typical punctate ERES pattern (Additional file 7: Figure S7). These GFP-Sec23B-positive ribbon-like structures were juxtaposed to Sec31A signals in normal condition as well as during elevated autophagy. These observations indicate the unlikely involvement of Sec23B in autophagy-induced response at the ERES.

On the Sec23A, the regions responsible for the interaction between Sec23 and Sec24 are from residues 160 to 200 and from 240 to 250 [34]. Serine 207 lies in a stretch of amino acids flanked by these regions. Ideally, phosphorylation at S207 may regulate Sec23-Sec24 interaction but this was not observed. We suspect that S207 phosphorylation may cause other changes. S207 is in a non-structural region in trunk domain. Phosphorylation at this site may cause change at secondary structure of this region and confer its effect during autophagy. On the other hand, threonine 405 is located within the β-barrel domain of Sec23 and this region is involved in interacting with Sec31 together with Sar1 [34, 35]. However, we observed a drastic decreased affinity for Sec31A by T405A (Fig. 5a). This is unexpected because ULK1 phosphorylation reduced the interaction between Sec23A and Sec31A. The phospho-mimicking T405D, however, was highly punctate at the ERES and thus, recapitulated the increase in ERES fluorescence as we predicted. We think that mutating T405 may have caused structural change that does not faithfully mimic the conformational change by phosphorylation. Nonetheless, it is difficult to ignore the role of T405 in Sec31A and Sar1 binding. When double mutants of these residues were created, all of the mutants show drastic reduction of Sec31A binding (Fig. 6c), further suggesting that T405 mutation may cause structural change to the Sec23A protein. This problem is most obvious in S207A,T405A double mutant, whose protein expression was drastically lower than others because of protein misfolding and degradation. These structural changes caused by mutagenesis prevented us from a more informative assessment of the functional consequences of ULK1 phosphorylation of Sec23A. Nonetheless, regulation of ER export by autophagy, via the action of ULK1, is consistent with the increasing evidence of phospho-regulation of COPII coat components as a means of controlling cellular secretion [26, 33], a process once thought to happen constitutively.

## Conclusions

ULK1 phosphorylates Sec23A at residues serine 207 and threonine 405. Serine 207 phosphorylation reduces the interaction between Sec23A and Sec31A and thus, reduces cargo export at the ERES and causes morphological changes at this organelle.

## Additional files

Additional file 1: Figure S1. Aggregation of ERES fluorescence puncta during active autophagy. Boxed areas of the pericentriolar ERES from cells grown in growth medium (left) and amino acid starved medium (right) were plotted as a function of the XY position of each pixel from the boxed area. The number of signal peaks was reduced in amino acid starved medium but the size of the peaks was larger, indicating aggregation of ERES signals into fewer and brighter fluorescence puncta. (TIFF 4537 kb)

Additional file 2: Figure S2. Intense ERES puncta is reversible. (A) Re-introduction of growth medium after amino acid starvation could reverse the ERES morphology back to normal. (B) Autophagy inhibitor MHY1485 subdued the autophagy-induced ERES morphology. (TIFF 3530 kb)

Additional file 3: Figure S3. General secretion is inhibited by elevated autophagy. Hela cells stably transfected with secreted alkaline phosphatase (SEAP) were used in this experiment. The enzymatic activity of SEAP served as a marker for evaluating protein secretion. Autophagy was activated by either supplementing rapamycin in culture medium or by amino acid starvation. The secretion of SEAP into the culture medium was determined for SEAP activity after 6 and 24 h. The SEAP activities were normalized by the total SEAP activities of the cell lysates. The ratio of SEAP activities from three independent experiments were determined and shown. Error bars = S.D. (TIFF 1339 kb)

Additional file 4: Figure S4. Transport marker GFP-VSVG-tsO45 accumulated in the ERES during elevated autophagy in HEK293 cells. VSVG tsO45 was accumulated at the ER at the ER at 39.5 °C before chase and then chased out of the ER for 10 and 30 min at 32 °C. VSV-G signals stayed in the ER exit sites 10 and 30 min after temperature was shifted to 32 C in cells undergoing active autophagy (by amino acid starvation (indicated as AA starv.) (arrows, upper panels, 10 and 30 min). In cells with growth medium, all the VSV-G signals have moved to the Golgi (G) at these time points (arrows, lower panels, 10 and 30 min). Quantitation of the fluorescence puncta of peripheral ERES having VSV-G signals was done in cells at 30 min after 32 °C incubation and was presented in lower right panel. Counting of the GFP-VSVG signals colocalized with peripheral ERES puncta was easier to be determined than the signals associated with the pericentriolar regions. Approximately 400 fluorescence dots from at least 8 cells for each condition were analyzed; Error bar = S.D. (TIFF 7535 kb)

Additional file 5: Figure S5. Sec23A mutants in COS cells. The indicated Myc-His-Sec23A mutants were transfected into COS cells before fixation and staining with anti-c-Myc antibody. (TIFF 3776 kb)

Additional file 6: Figure S6. The interactions between the ULK1 phosphorylation mutants of Sec23A and Sec31A. (A) S207 and T405 mutants of Sec23A were tested for their interaction with Sec31A by co-expressing the Myc-His-Sec23A mutants and GFP-Sec31A. (B) Combinations of S207 and T405 double mutants were tested for the interaction with Sec31A. Overexpression of the indicated protein was carried in HEK293T cells, followed by immunoprecipitation of Myc-His-Sec23A by anti-Myc antibody. Co-precipitated GFP-Sec31A was detected by anti-GFP antibody. (B) Wildtype Sec23A, S207A and T405A mutants were tested for the interaction with GFP-Sec31A in growth medium or in amino acid starved medium EBSS. (TIFF 5305 kb)

Additional file 7: Figure S7. GFP-Sec23B was poorly localized with Sec31A positive ERES. GFP-Sec23B (green) and Sec31A (red) were visualized in Hela cells grown in growth medium or after amino acid starvation for 1 and 2 h. GFP-Sec23B signal were largely ribbon-like structures juxtaposed Sec31A signals. In cells in which GFP-Sec23B signals were punctate, colocalization with Sec31A was still poor (inset). (TIFF 5601 kb)

## Abbreviations

ER: Endoplasmic reticulum
ERES: ER exit sites
GFP: Green fluorescent protein
HRP: Horse radish peroxidase
PAS: Preautophagosomal structure
SEAP: Secreted alkaline phosphatase
TRAPP: Transport protein particle
VSVG: Vesicular Stomatitis Virus G glycoprotein
