# Supplementary figures and images for: ULK1 phosphorylates Sec23A and mediates autophagy-induced inhibition of ER-to-Golgi traffic
Source: BMC Cell Biol. 2017 May 10;18:22. doi: 10.1186/s12860-017-0138-8 (PMC5424413; doi:10.1186/s12860-017-0138-8)

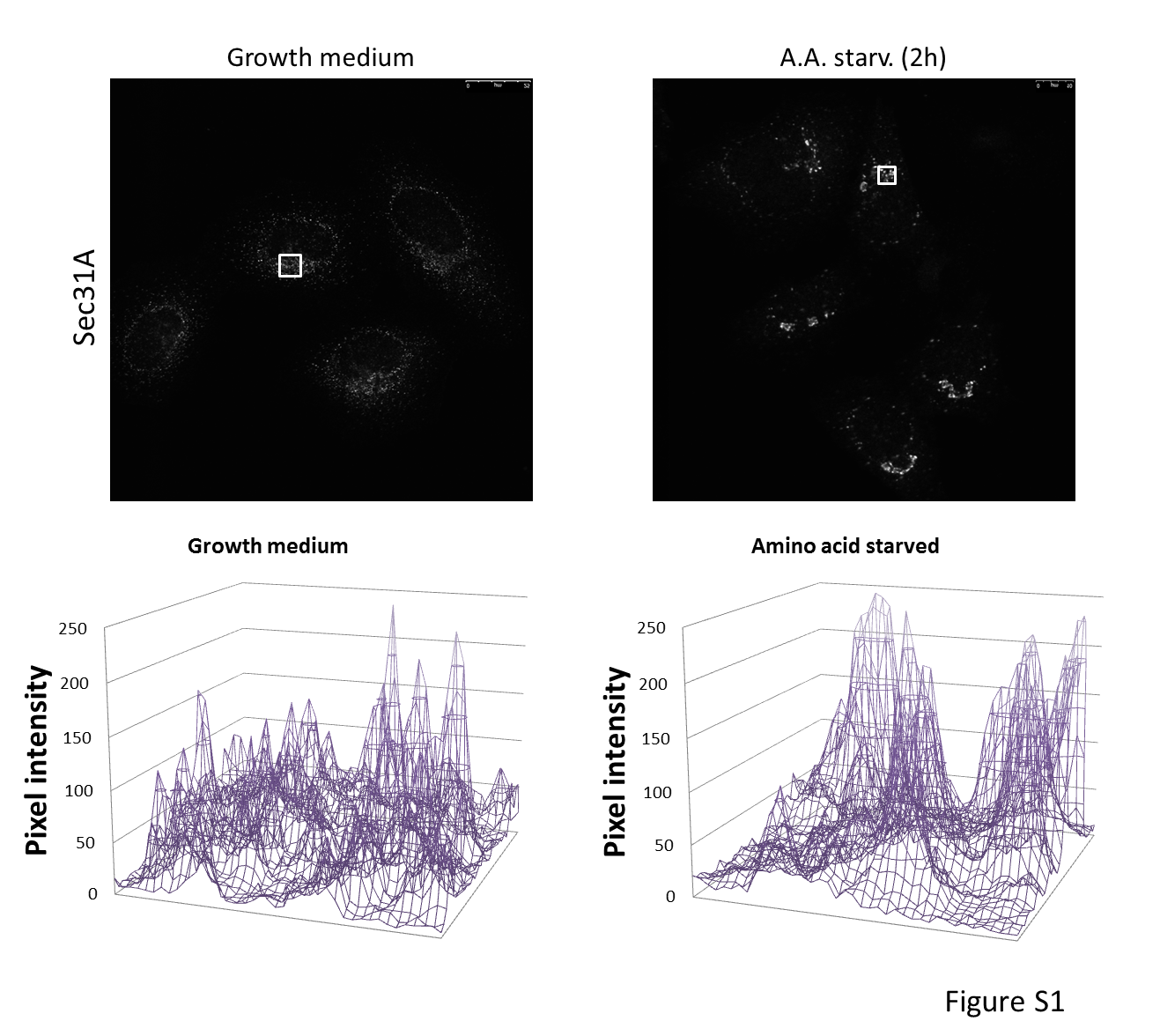

Supplement: Supplementary file 1 — Aggregation of ERES fluorescence puncta during active autophagy. Boxed areas of the pericentriolar ERES from cells grown in growth medium (left) and amino acid starved medium (right) were plotted as a function of the XY position of each pixel from the boxed area. The number of signal peaks was reduced in amino acid starved medium but the size of the peaks was larger, indicating aggregation of ERES signals into fewer and brighter fluorescence puncta. (TIFF 4537 kb) [file 12860_2017_138_MOESM1_ESM.tif]

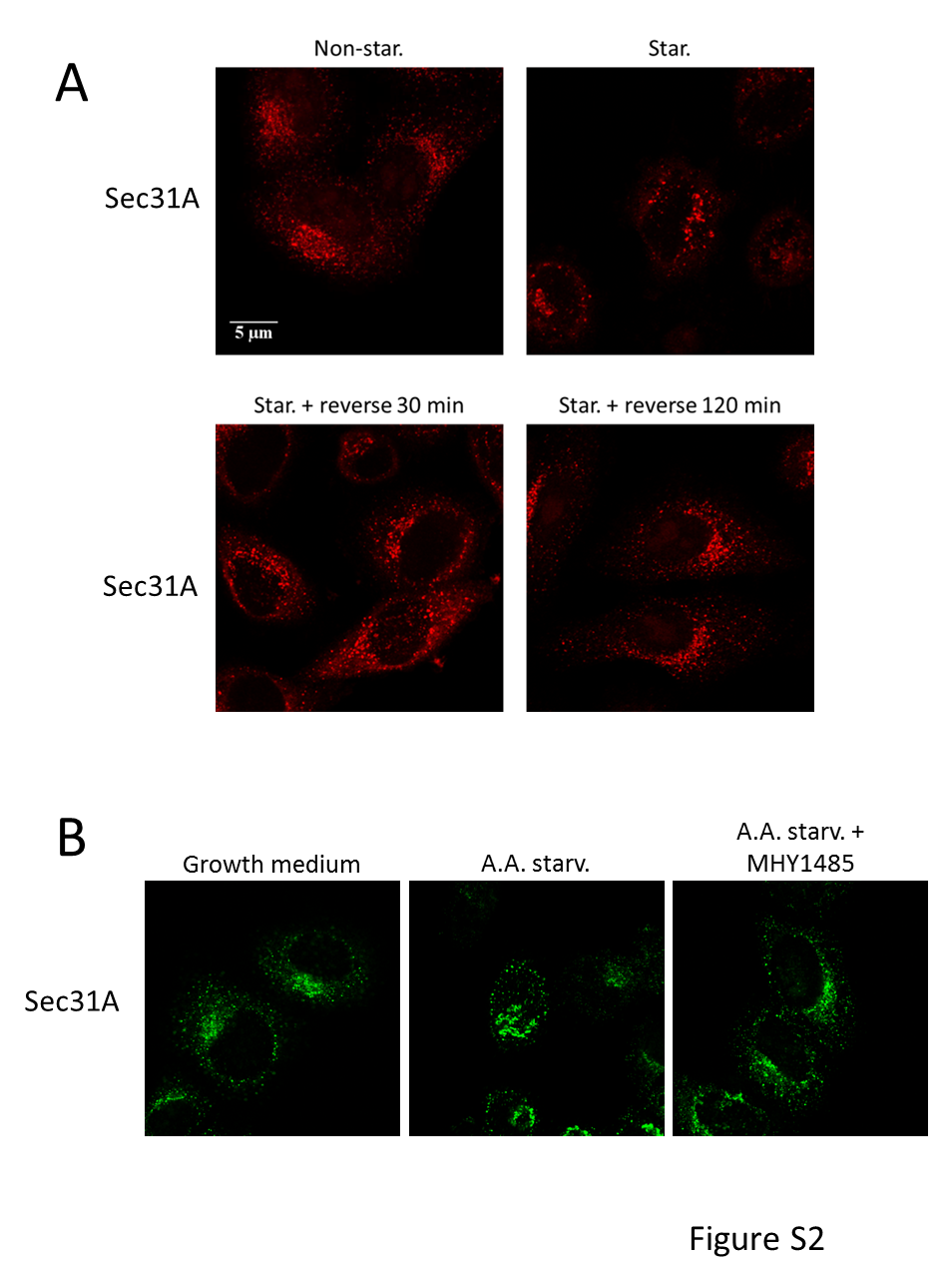

Supplement: Supplementary file 2 — Intense ERES puncta is reversible. (A) Re-introduction of growth medium after amino acid starvation could reverse the ERES morphology back to normal. (B) Autophagy inhibitor MHY1485 subdued the autophagy-induced ERES morphology. (TIFF 3530 kb) [file 12860_2017_138_MOESM2_ESM.tif]

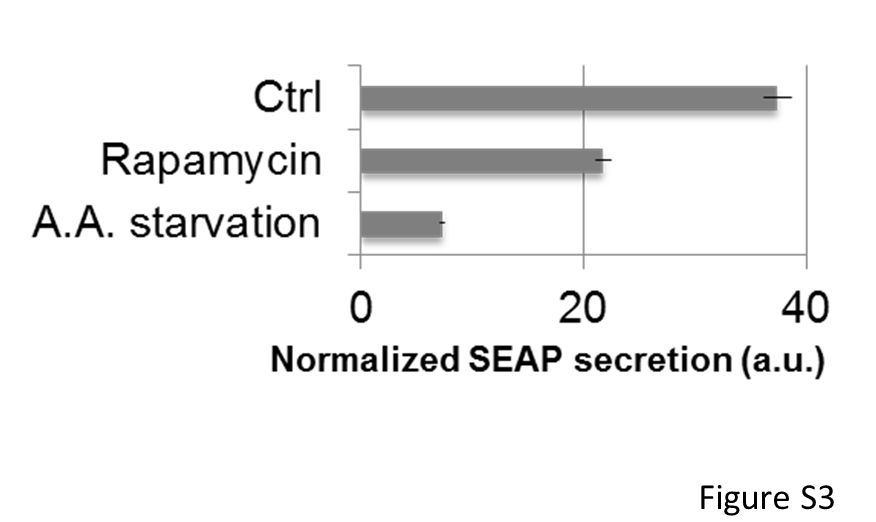

Supplement: Supplementary file 3 — General secretion is inhibited by elevated autophagy. Hela cells stably transfected with secreted alkaline phosphatase (SEAP) were used in this experiment. The enzymatic activity of SEAP served as a marker for evaluating protein secretion. Autophagy was activated by either supplementing rapamycin in culture medium or by amino acid starvation. The secretion of SEAP into the culture medium was determined for SEAP activity after 6 and 24 h. The SEAP activities were normalized by the total SEAP activities of the cell lysates. The ratio of SEAP activities from three independent experiments were determined and shown. Error bars = S.D. (TIFF 1339 kb) [file 12860_2017_138_MOESM3_ESM.tif]

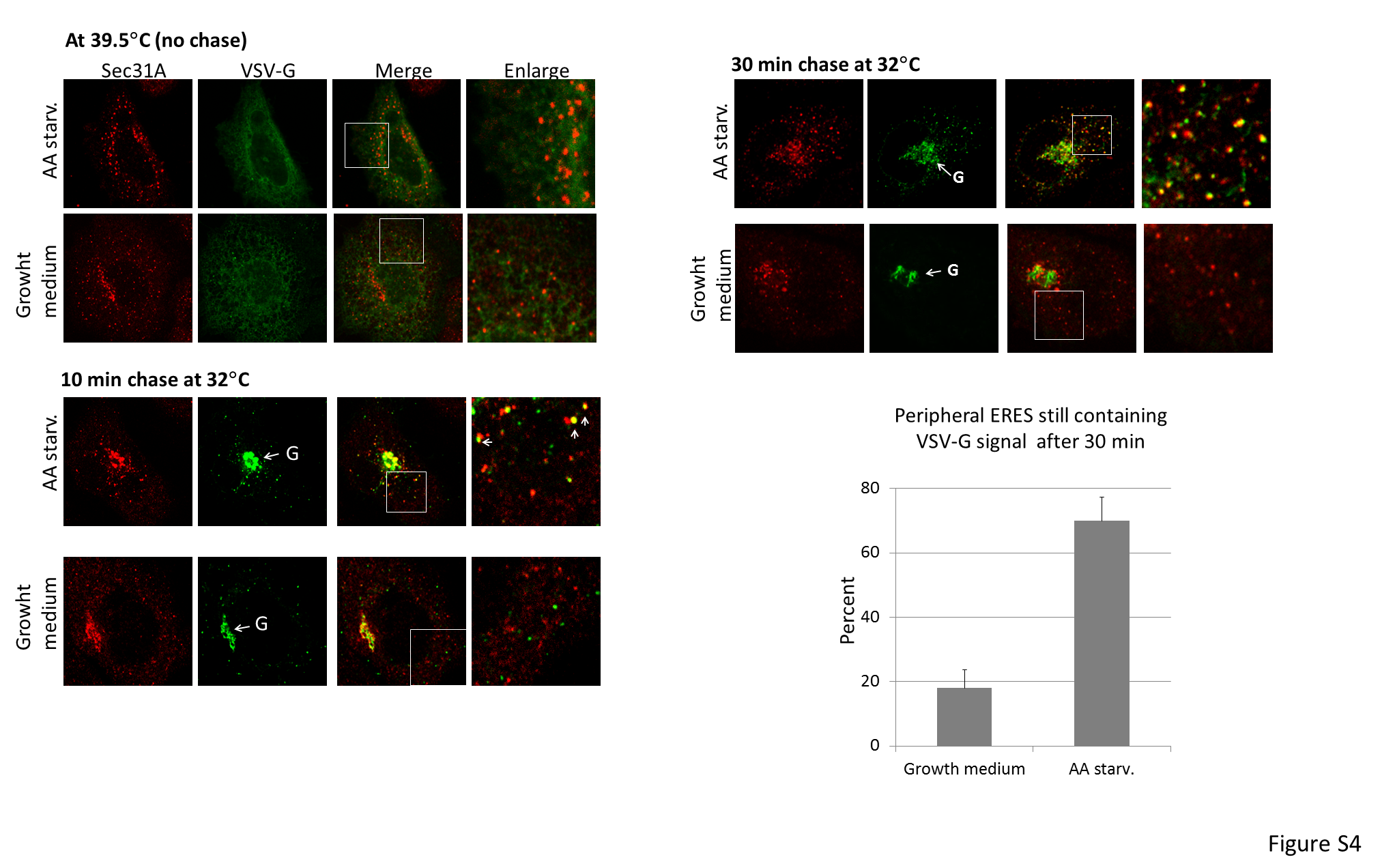

Supplement: Supplementary file 4 — Transport marker GFP-VSVG-tsO45 accumulated in the ERES during elevated autophagy in HEK293 cells. VSVG tsO45 was accumulated at the ER at the ER at 39.5 °C before chase and then chased out of the ER for 10 and 30 min at 32 °C. VSV-G signals stayed in the ER exit sites 10 and 30 min after temperature was shifted to 32 C in cells undergoing active autophagy (by amino acid starvation (indicated as AA starv.) (arrows, upper panels, 10 and 30 min). In cells with growth medium, all the VSV-G signals have moved to the Golgi (G) at these time points (arrows, lower panels, 10 and 30 min). Quantitation of the fluorescence puncta of peripheral ERES having VSV-G signals was done in cells at 30 min after 32 °C incubation and was presented in lower right panel. Counting of the GFP-VSVG signals colocalized with peripheral ERES puncta was easier to be determined than the signals associated with the pericentriolar regions. Approximately 400 fluorescence dots from at least 8 cells for each condition were analyzed; Error bar = S.D. (TIFF 7535 kb) [file 12860_2017_138_MOESM4_ESM.tif]

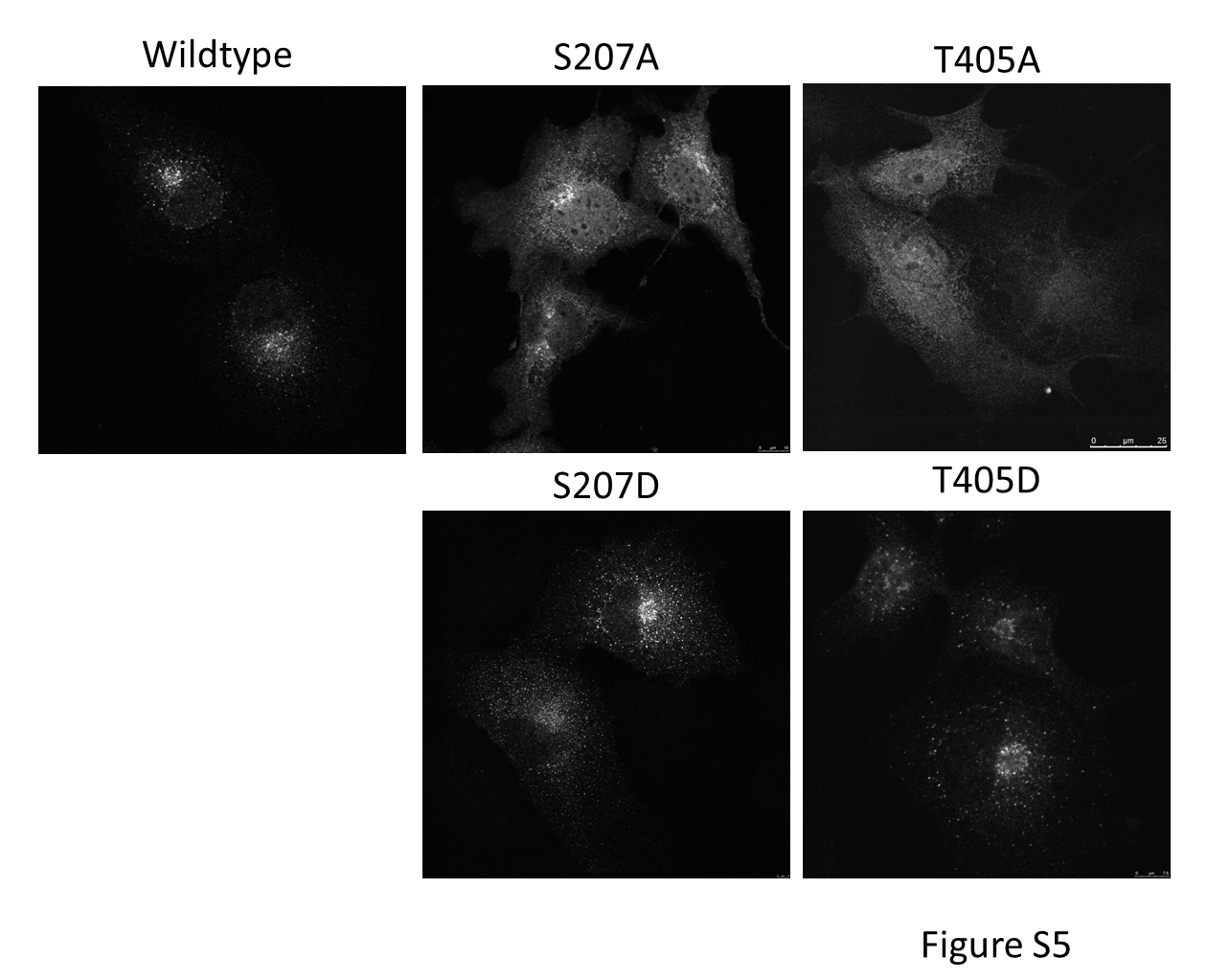

Supplement: Supplementary file 5 — Sec23A mutants in COS cells. The indicated Myc-His-Sec23A mutants were transfected into COS cells before fixation and staining with anti-c-Myc antibody. (TIFF 3776 kb) [file 12860_2017_138_MOESM5_ESM.tif]

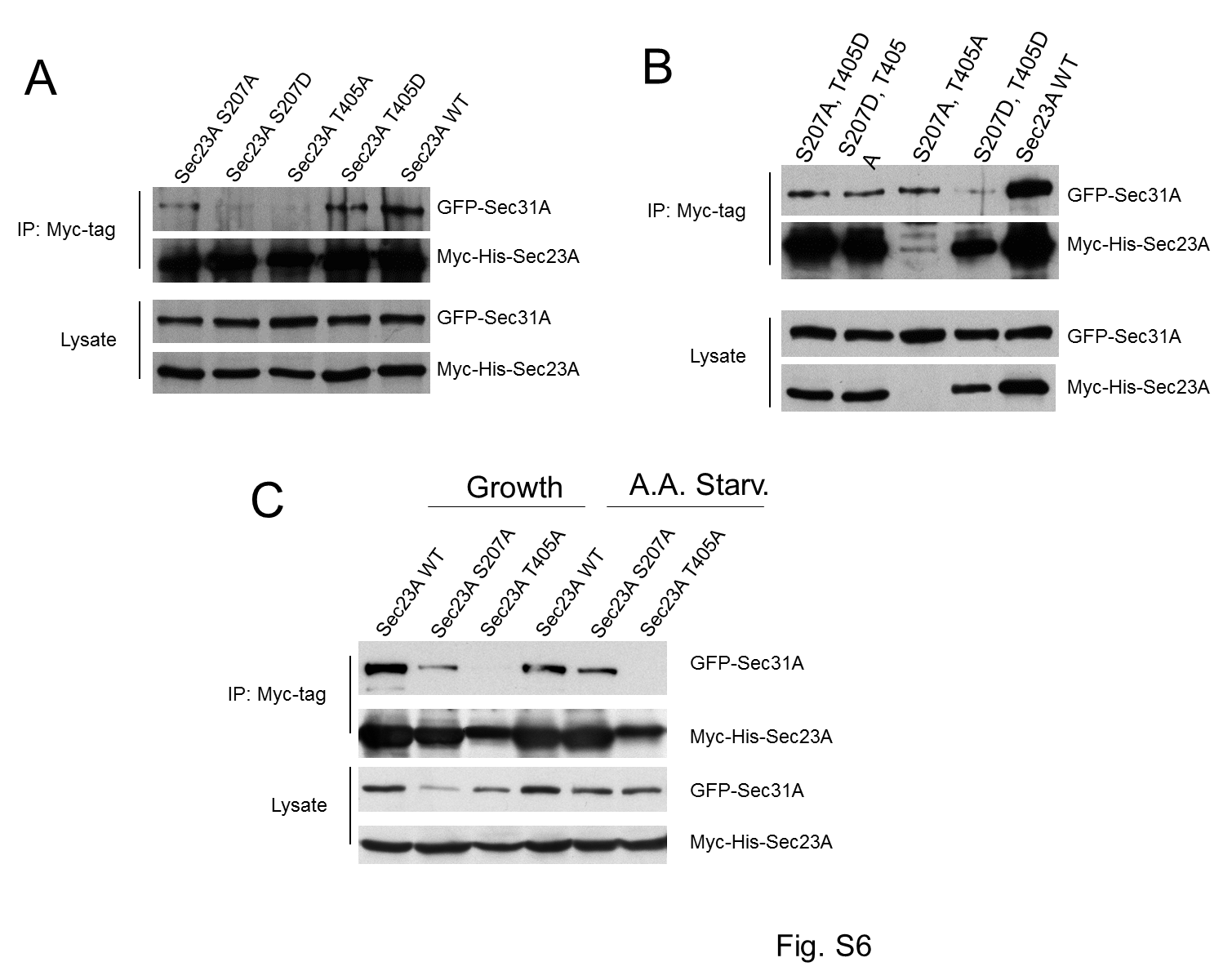

Supplement: Supplementary file 6 — The interactions between the ULK1 phosphorylation mutants of Sec23A and Sec31A. (A) S207 and T405 mutants of Sec23A were tested for their interaction with Sec31A by co-expressing the Myc-His-Sec23A mutants and GFP-Sec31A. (B) Combinations of S207 and T405 double mutants were tested for the interaction with Sec31A. Overexpression of the indicated protein was carried in HEK293T cells, followed by immunoprecipitation of Myc-His-Sec23A by anti-Myc antibody. Co-precipitated GFP-Sec31A was detected by anti-GFP antibody. (B) Wildtype Sec23A, S207A and T405A mutants were tested for the interaction with GFP-Sec31A in growth medium or in amino acid starved medium EBSS. (TIFF 5305 kb) [file 12860_2017_138_MOESM6_ESM.tif]

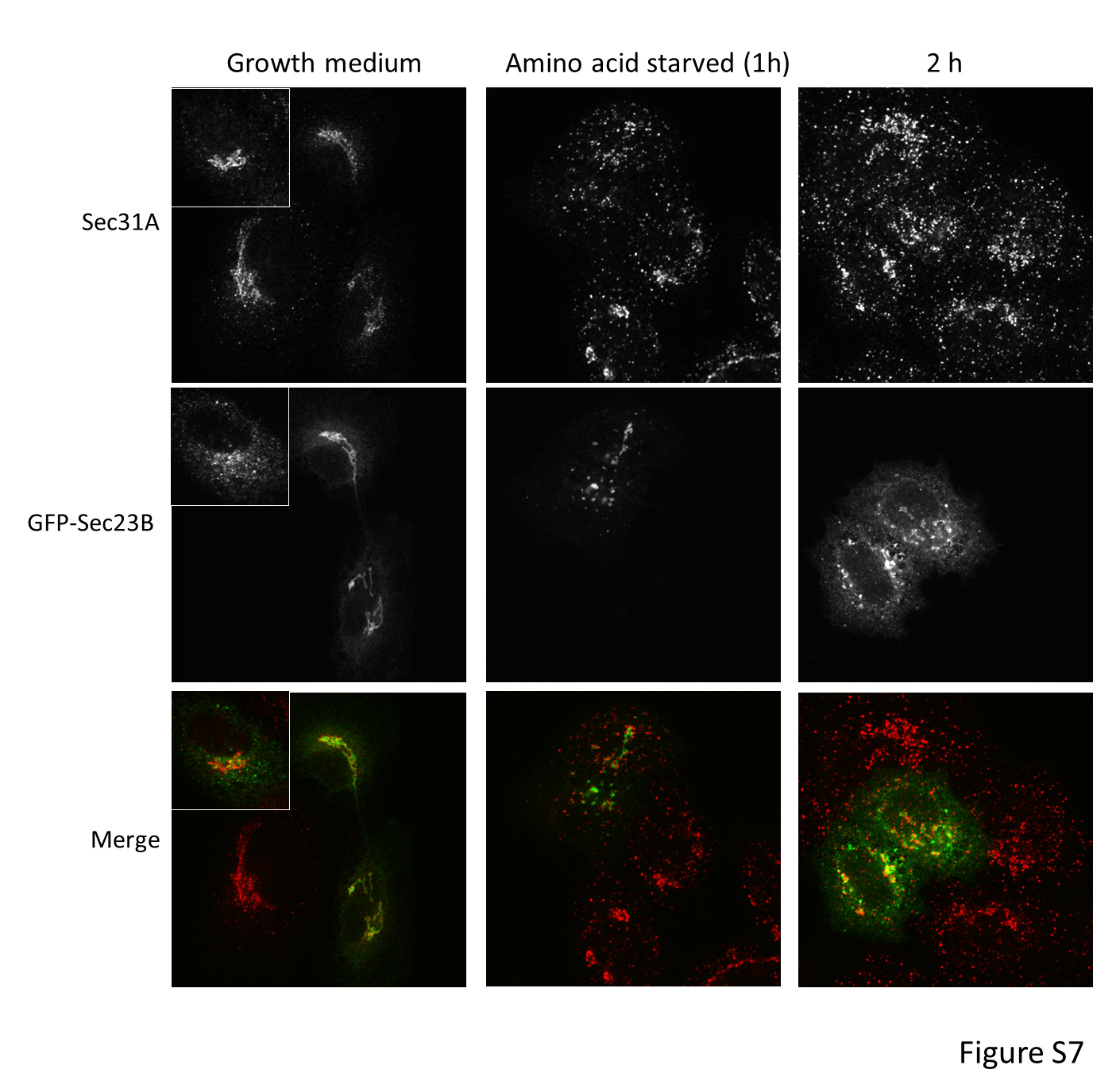

Supplement: Supplementary file 7 — GFP-Sec23B was poorly localized with Sec31A positive ERES. GFP-Sec23B (green) and Sec31A (red) were visualized in Hela cells grown in growth medium or after amino acid starvation for 1 and 2 h. GFP-Sec23B signal were largely ribbon-like structures juxtaposed Sec31A signals. In cells in which GFP-Sec23B signals were punctate, colocalization with Sec31A was still poor (inset). (TIFF 5601 kb) [file 12860_2017_138_MOESM7_ESM.tif]
